# Can the Vocal Expression of Intellectually Disabled Individuals Be Used as a Pain Indicator? Initial Findings Supporting a Possible Novice Assessment Method

**DOI:** 10.3389/fpsyg.2021.655202

**Published:** 2021-07-22

**Authors:** Michal Icht, Hadar Wiznitser Ressis-tal, Meir Lotan

**Affiliations:** ^1^Department of Communication Disorders, Ariel University, Ariel, Israel; ^2^Holistic Physical Therapy Private Clinic, Jerusalem, Israel; ^3^Department of Physiotherapy, Ariel University, Ariel, Israel

**Keywords:** intellectual and developmental disability, pain, voice, vocal characteristics, acoustical analysis

## Abstract

Pain is difficult to assess in non-verbal populations such as individuals with intellectual and developmental disability (IDD). Due to scarce research in this area, pain assessment for individuals with IDD is still lacking, leading to maltreatment. To improve medical care for individuals with IDD, immediate, reliable, easy to use pain detection methods should be developed. The goal of this preliminary study was to examine the sensitivity of acoustic features of vocal expressions in identifying pain for adults with IDD, assessing their feasibility as a pain detection indicator for those individuals. Such unique pain related vocal characteristics may be used to develop objective pain detection means. Adults with severe-profound IDD level (*N* = 9) were recorded in daily activities associated with pain (during diaper changes), or without pain (at rest). Spontaneous vocal expressions were acoustically analyzed to assess several voice characteristics. Analyzing the data revealed that pain related vocal expressions were characterized by significantly higher number of pulses and higher shimmer values relative to no-pain vocal expressions. Pain related productions were also characterized by longer duration, higher jitter and Cepstral Peak Prominence values, lower Harmonic-Noise Ratio, lower difference between the amplitude of the 1st and 2nd harmonic (corrected for vocal tract influence; H1H2c), and higher mean and standard deviation of voice fundamental frequency relative to no-pain related vocal productions, yet these findings were not statistically significant, possibly due to the small and heterogeneous sample. These initial results may prompt further research to explore the possibility to use pain related vocal output as an objective and easily identifiable indicator of pain in this population.

## Introduction

Intellectual and developmental disability (IDD) results from a variety of disorders, including congenital brain malformations, brain injuries, and other genetic or acquired syndromes. The prevalence of IDD is close to 3%, while the estimated prevalence of severe and profound IDD is about 0.1% of the population (Maulik et al., 2011). Studies in this field indicate that people with IDD have 2.5 times more health problems than people without IDD (Van Schrojenstein Lantman-De Valk et al., 2000). Specifically, individuals with severe and profound IDD often have sensory impairments and physical health problems (e.g., dysphagia, epilepsy, spasticity, and reflux disease), negatively impacting all aspects of the quality of life (Van Timmeren et al., 2016).

Available findings regarding pain in individuals with severe IDD suggest that pain in this population is common, yet rarely actively treated (Stallard et al., 2001). Individuals with severe or profound levels of IDD are more likely to have additional disabling conditions or multiple complex medical problems coupled with communication difficulties. Such medical problems, whether directly or indirectly linked to the disability, often necessitate painful procedures, including physical therapy treatments and various medical interventions. Moreover, individuals with severe cognitive impairments and low communication abilities are likely to experience the most pain over time (Breau et al., 2003).

A study investigating the frequency, duration, intensity, and location of pain, as well as the interference of pain with activities, in adults with cerebral palsy (CP) and IDD, found that pain was a significant problem for the majority of participants (Schwartz et al., 1999). Of the 93 participants, one or more areas of chronic pain (minimum of three months’ duration) was reported by 67% of the participants, and 53% experienced moderate to severe pain on an almost daily basis. The mean duration of pain lasted 20 years suggesting a long-term severe predicament. Likewise, McGrath et al. (2000) investigated the pain experience of 64 children with IDD and found that they suffered pain on a regular basis, with 83% suffering constant pain at a level higher than three on a 10-point scale.

It is clear that better pain management should start with proper pain evaluation and that it is essential for the clinician to use reliable evaluation tools to initiate the pain assessment and intervention processes. Despite such understanding, assessing pain in individuals with IDD is a challenging task and can become extremely difficult at the levels of severe and profound IDD, where the ability to verbally communicate pain experience can be severely compromised (Lachapelle et al., 1999). Without objective assessment, pain can be misinterpreted or underestimated, which might lead to inadequate management and undermine quality of life (Malviya et al., 2001).

Given the constant hindrance of pain to quality of life among individuals with IDD, there is an urgent need to develop a proper pain assessment tool for this population. Yet, the scientific world has lagged behind when it comes to pain assessment in individuals with IDD, and there are several reasons for this situation. First, many individuals with IDD have neurological problems that may affect their ability to effectively and clearly communicate their pain, thus complicating evaluation of the qualitative and quantitative aspects of their pain experience (Oberlander et al., 1999). These communication problems make it difficult for individuals with IDD to respond to questions about their pain, for example, they may respond in a way that is not meaningful to caregivers (Breau et al., 2004). These circumstances make pain deciphering within conventional means in these patients, highly difficult or in some cases impossible (Mafrica et al., 2006).

Second, individuals with IDD often present unclear communicative signals, as well as present challenging behaviors such as aggression, self-injury, and tantrums (Carr and Owen-DeSchryver, 2007), which might mask pain signals (Clements, 1992). This makes it difficult to ascertain whether the observed behavior is attributable to pain or another source of distress or whether it is simply part of the individual’s regular aberrant behavior.

Thirdly, behavioral indicators of pain in the general population, such as facial grimaces, groaning, or altered sleep patterns (Bodfish et al., 2001), may well appear in individuals with IDD at times when they are not in pain (McGrath et al., 1998). It is therefore not surprising that such behaviors are attributed to the intellectual level of the individual rather than to pain (Mason and Scior, 2004), again resulting in under-diagnosing of pain.

Finally, assessing and managing pain in people with IDD can be complicated by the effects of medication (Turk and Melzack, 2001), as well as the lack of appropriate pain assessment tools. Despite the increased research attention focused on expressive behavior related to pain in individuals with IDD (Carter et al., 2002; Donovan, 2002; Fanurik et al., 1999; Stallard et al., 2001; Stallard et al., 2002a), research on this topic is still scarce and there are but few pain-assessment scales available for use in this specific population.

Most existing pain scales for this population such as the Facial Action Coding System [FACS; (Ekman and Friesen, 1978)], the Evaluation Scale for Pain in Cerebral Palsy [ESPCP; (Giusiano et al., 1995)], the Non-Communicating Children’s Pain Checklist [NCCPC; (Breau et al., 2000)], the Pain Indicator for Communicatively Impaired Children [PICIC; (Stallard et al., 2002b)], Pediatric Pain Profile [PPP; (Hunt et al., 2004)], and the Pain and Discomfort Scale [PADS; (Bodfish et al., 2001)] are behavioral scales relying on proxy report necessitating previous acquaintance with the person being evaluated. The downside of proxy reports is that it can be misinterpreted by health care professionals (American Academy of Pediatrics Committee on Psychosocial Aspects of Child and Family Health, and Task Force on Pain in Infants, Children, and Adolescents, 2001), as well as by relatives (Britto et al., 2004; Ennett et al., 1991; Schwartz and Rabinovitz, 2003). Therefore, there is a need to examine novice objective measures which might present an alternative, perhaps a more objective measure, to existing pain assessment scales.

One of these possibilities is the use of vocalizations as communicative signals, and specifically as expressions of pain (Lautenbacher et al., 2017; Scheiner et al., 2006). When experiencing pain, individuals produce pain related vocalizations, such as non-verbal cries or screams (Fuller et al., 1993). Hence, vocalizations are considered indicators of pain in individuals unable to provide reliable self-reports concerning their subjective pain experience, such as older adults with advanced dementia (Herr et al., 2011; van Iersel et al., 2006). The literature reports that pain can be recognized and even classified (according to levels of intensity) through vocal or acoustic cues. For example, increases in the mean and range of voice fundamental frequency (F0), the amplitude and the degree of periodicity of the vocalization were associated with increased level of simulated pain intensity, and predicted increases in listeners’ ratings of pain intensity (Raine et al., 2019).

Few studies have addressed the possibility to evaluate pain in non-verbal individuals with IDD using their vocal expressions. McGrath et al. (1998) analyzed caregivers’ interviews and found that most individuals (80–90%) displayed pain related vocal behaviors such as moaning, whining, and crying in a moderately loud intensity. Similar findings were reported by Carter, McArthur, and Cunliffe (Carter et al., 2002), as parents of children with profound special needs often considered vocalization (mainly unique, high pitched cry or moan) a key element of pain expression. Interestingly, pain vocalization was described as being qualitatively different from other cries (e.g., those associated with frustration or distress), and was also found to differ between non-verbal children with IDD and their typically developing (TD) peers (Dubois et al., 2010). The vast agreement between caregivers and parents regarding specific recognizable vocal expression as pain signals points out the possibility of using vocal acoustic traits as a reliable signifier for this population.

Shedding further light on this issue, this exploratory study aimed to identify specific acoustic traits of pain-related vocalizations relative to no-pain-related daily vocal productions of young adults with severe to profound level of IDD. Audio tracks recordings of daily experiences reflecting no-pain and painful situations according to the caregivers (using VAS) as well as by the researchers of a previous project (using NCAPC) were acoustically analyzed within the present investigation. The results may guide the development of an immediate, reliable, easy to use and objective pain detection methods.

## Materials and Methods

### Ethics

The original research evaluating pain in adults with IDD (Wiznitser, 2019) was approved by the Ariel University IRB, as well as by the medical director of the ministry of welfare. All parents or legal guardians of the participants have signed an acknowledged consent form approving the participation of their protegees as participants of this research project. Within the present research we acoustically analyzed non-identifiable audio recordings of individuals with IDD recorded while following the previous research protocol.

### Participants

The present article presents acoustical analysis extracted from data collected during a former research project aimed to estimate the frequency of pain due to hip dislocation among adults with severe to profound IDD (Wiznitser, 2019).

In the former study, 30 adults with a diagnosis of severe-profound level of IDD and a secondary diagnosis of a congenital hip dislocation participated. All participants were highly dependent on the support of caregivers to participate in any routines or activities, including communication. All participants were non-verbal and showed a very limited independent ability to use language or symbols in any form. They rarely initiate communication and communicate in restricted and inconsistent ways.

### Study Protocol

All 30 participants were videotaped once before and once during acute pain (daily activities including movement of the legs, e.g., diaper change, a painful procedure due to the congenital hip dislocation), using a hand-held Sony video camera (Digital 8 Megapixel), with Electret Condenser built-in microphone technology. Specifications of the Audio recording system: Rotary heads, PCM system. Quantization: 12 bits (Fs 32 kHz, stereo 1, stereo 2), 16 bits (Fs 48 kHz, stereo). For each participant, two recordings were obtained, “pain” (during diaper change) and “no pain” (at rest), a total of 60 recordings.

As part of the former study, the video recordings were analyzed by two caregivers, separately, to evaluate pain behaviors, using the following tools: (a) The Non-Communicating Adult’s Pain Checklist (NCAPC) – The NVAPC was developed by the third author (ML) to identify pain behaviors of adults with IDD (Lotan et al., 2002; Lotan et al., 2009a). It is an 18-item scale which was found sensitive to pain with very good psychometric values (Lotan et al., 2009a,b) presenting a theoretical model of pain for this population (Lotan et al., 2012), as well as showing clinical feasibility (Weissman–Fogel et al., 2015), and (b) Visual Analogue scale (VAS) – The scale is a validated, subjective measure for acute and chronic pain. Scores are recorded by making a handwritten mark on a 10-cm line that represents a continuum between “no pain” and “worst pain” (Abou El–Soud and El Shenawy, 2010). Caregivers used this scale to express their opinion regarding daily pain experience of the participants. Both tools showed high inter-rater reliability (NCAPC ICC = 0.92; VAS ICC = 0.97).

### Voice Samples

Of the original 60 video recordings (of 30 participants), 18 recordings (“pain” recording and “no-pain” recording of nine participants) were clear enough to allow the current acoustical analysis (i.e., comprise detectable voice signals, not corrupted with background noise or other interfering signals). The final dataset included those 18 audio recordings, voice samples of four males and five females; mean age: 26:6 years, SD: 6:2 years (participants’ information is presented in Table 1).

**TABLE 1:** Participants’ data.

**No.**	**Gender**	**Etiology; Related characteristics**
1	F	CP; Spastic quadriplegia
2	M	CP; Spastic quadriplegia
3	F	Chromosomal abnormalities
4	M	CP; Spastic quadriplegia
5	F	CP; Spastic quadriplegia; Severe brain damage S/P Neonatal asphyxia
6	F	CP; SGA; S/P Preterm 29w
7	F	CP; Spastic quadriplegia
8	M	CP; Spastic quadriplegia
9	M	Severe brain damage

The repertoire of nonverbal communicative displays included various call types, mostly vowel-like, for example, moaning, crying, and yelling, for both pain, and non-pain productions.

### Audio Recordings

The audio contents of the digital video recordings were extracted to provide 16 bits at 44.1 kHz sampling rate WAV files. The original video recordings contained several audio signals (e.g., participants’ vocalizations, caregiver speech, silence, environmental noise). Therefore, as a first step we removed irrelevant signals and long silent regions using the SoundForge program. Then, the 18 voice samples (“pain” and “no-pain” recordings of nine participants) were stored in the computer and analyzed.

### Acoustical Analysis

**FIGURE 1:**
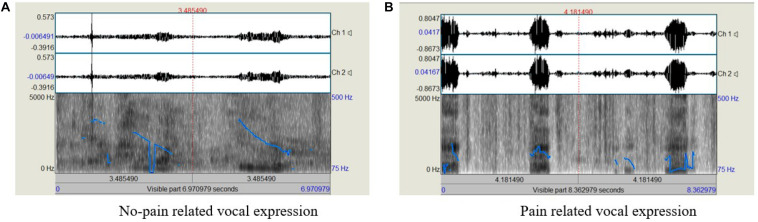
Examples of the acoustic analysis of the voice recordings; **(A)** No-pain related sample, with shorter overall duration and fewer pulses, **(B)** pain-related sample, with longer duration and more pulses.

Acoustic analyses of the voice samples were performed using Praat software (Boersma and Weenink, 2020). Some examples of the voice recordings used are shown in Figure 1. Following the removal of irrelevant audio signals, in each recording we focused on participants’ vocalizations (each pulse or interval within the recording) by selecting the boundaries of the pulses (positioning the PRAAT cursor; from the beginning, or onset, of phonation, to the end, or offset, of phonation). The relevant information was then extracted as an average of the marked segment.

Each voice sample was analyzed (means and standard deviations, *SD*s) for (a) the duration of the utterance - calculated for this measure as the total duration of the voiced segments in each recording (from the beginning to the end of phonation), measured in seconds, (b) number of pulses, or segments in which a clear phonation (harmonic structure) can be detected within the utterance, (c) jitter - the parameter of frequency variation from cycle to cycle (%), (d) shimmer – the parameter of the amplitude variation from cycle to cycle (%), (e) Harmonic-Noise ratio (HNR) – the ratio of the acoustic energy of the harmonic components to that of the noise (dB), (f) Cepstral Peak Prominence (CPP) – another measure of the degree of harmony within a voice sample, obtained using a Fourier transform of the logarithm power spectrum (dB), (g) H1H2c – measures the difference in amplitude between the first two harmonics (a measure of spectral tilt, which is highly correlated with the degree of glottal constriction, dB), and (h) mean and *SD*s of the fundamental frequency (F0) – the number of cycles of opening/closure of the glottis (Hz).

The mean and *SD* of F0 were measured using Praat. Minimum pitch was set to 50 Hz and maximum pitch to 500 Hz, which covers the range of both male and female voices and is suitable for the type of vocalizations analyzed. Note, pitch ranges should be different between female and male voices but because we included individuals with severe to profound IDD level, who may not present as typical female and male voices, we chose to use a relatively wide range, in accord with the recommendations listed in the PRAAT-Manual [(van Lieshout, 2017); See also: (Alhinti et al., 2021; Lee et al., 2009; Patel, 2003)].

CPP and H1H2c were obtained using PraatSauce, a Praat-based application for spectral measures based on VoiceSauce (Kirby, 2018). H1H2 measure was corrected for formant frequencies and bandwidths, to account for the fact that formants raise the amplitudes of nearby harmonics, making it difficult to compare H1–H2 values across different productions (Hanson et al., 2001). These measures were extracted from 10 equidistant points from the marked segment, with an average of the middle eight points used for analysis.

Note, as the distance between the participants’ mouth and the video camera was not controlled, voice intensity (loudness) was not analyzed. To avoid possible biases, and to ensure the accuracy of cursor position and the selection of boundaries, the analyses were conducted by two trained research assistants, Speech-Language Pathology undergraduates trained in acoustical analysis, who were blinded to the experimental condition. When the two did not agree on a specific sample (less than 5%, indicating strong interrater reliability), it was re-analyzed by an experienced SLP (the first author, MI).

### Statistical Analyses

Statistical analyses were carried out using SPSS-19 software (SPSS Inc., Chicago, Illinois). Statistical significance between pain and no-pain recordings for the following acoustic measures: duration, pulses, jitter, shimmer, HNR, CPP, and H1H2c, was determined using an exact Wilcoxon Signed-Rank Test, a non-parametric test used to compare two repeated measurements on a single sample that can be used as an alternative to the paired samples *t*-test when the distribution of the difference between the means cannot be assumed to be normally distributed (using a 0.05 probability level). Due to *a priori* gender differences in voice fundamental frequency, F0 data (mean, SD) were analyzed separately for female and male participants.

## Results

Table 2 lists the mean values (and SDs) of duration (in sec.), number of pulses, jitter (in %), shimmer (in %), HNR, CPP, H1H2c (in dB), and mean and SD of F0 (in Hz; separately for female and male participants), for pain- and no-pain related voice samples.

**TABLE 2:** Results – Mean (and *SD*s) for the various acoustic measures in pain and no-pain conditions.

**Acoustic measure**	**No pain**		**Pain**		***p***
Duration (sec)	0.93 (0.62)		1.09 (0.79)		NS
Pulses	2.66 (0.86)		4.11 (1.76)		0.026
Jitter (%)	1.81 (0.66)		2.47 (0.92)		NS
Shimmer (%)	11.15 (3.78)		14.36 (4.75)		0.048
HNR (dB)	13.02 (5.09)		10.80 (4.21)		NS
CPP (dB)	15.30 (2.20)		16.01 (1.86)		NS
H1-H2c (dB)	5.92 (5.66)		5.07 (4.50)		NS

	**Males^#^**	**Females**	**Males^#^**	**Females**	

F0 mean (Hz)	220.17 (108.5)	257.48 (89.44)	321.1 (203.7)	304.64 (96.74)	NS
F0 SD (Hz)	22.58 (17.21)	36.79 (26.66)	45.02 (38.67)	51.83 (48.57)	NS

*NS, non-significant. ^#^The sample size is too small to allow a reliable calculation of the exact Wilcoxon signed-rank statistic.*

Analyzing the data using the exact Wilcoxon signed-rank test, a significant difference was found in the number of pulses between pain related vocal productions (mean = 4.11, SD = 1.76) relative to no-pain related productions (mean = 2.66, SD = 0.86), *p* = 0.026. Similar analysis revealed a significant difference in the shimmer values between pain related productions (mean = 0.14, SD = 0.04) relative to no-pain related productions (mean = 0.11, SD = 0.03), *p* = 0.048.

**FIGURE 2:**
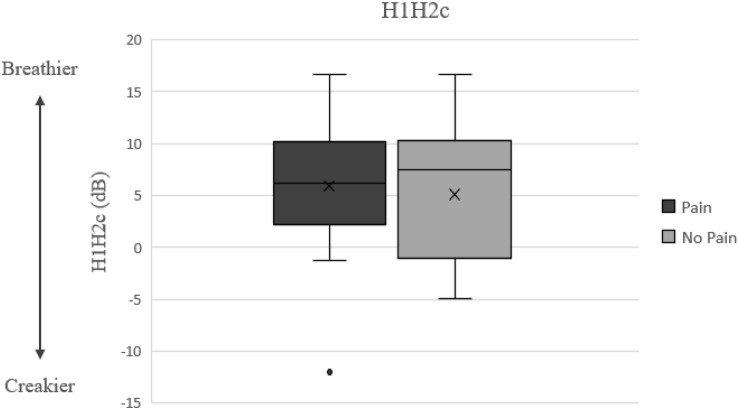
H1–H2c results and possible relationship to nonmodal phonation model [adapted from: (Gittelson et al., 2018)].

Observing the descriptive data in Table 2 also reveals that pain related productions were characterized by longer duration, higher jitter and CPP values, and lower HNR and H1H2c values (H1H2c results are visually depicted in Figure 2), and higher F0 mean and SD relative to no-pain related vocal productions, yet these differences were not statistically significant.

## Discussion

Individuals with severe IDD often suffer from pain due to chronic physical problems, complex medical disorders, and frequent injuries and medical procedures (Breau et al., 2003). However, their cognitive abilities and communicational restrictions, make it difficult to identify, detect and monitor their pain conditions. The present preliminary investigation set out to assess the possibility of using vocal expressions of non-verbal individuals with IDD as detectable pain signals, using acoustic analyses of pain related and no-pain related vocal productions. The main findings of the analyses showed that the number of pulses and shimmer values were significantly higher in pain related vocal productions relative to no-pain related productions. These initial results suggest that vocal monitoring might be a used as an objective method enabling pain detection in this population.

### Number of Pulses

Vocalization may be temporally characterized as continuous or segmented. Such rhythmic patterns characterize pain vocalization as well. In the current study, pain related vocal expressions were characterized by significantly higher number of tonal segments or pulses (4.11) relative to no-pain related vocal expressions (2.6), although the duration of pain and no-pain voice productions did not differ. These results can be compared to reports in another group of non-verbal individuals, namely infants. Indeed, the nonverbal vocalizations (call types) of these two groups, non-verbal adults with severe to profound level IDD and babies, share some vocal characteristics and are considered to be universal [for a discussion, see: (Anikin et al., 2018)]. For example, Porter et al. (1986) investigated the relation between neonatal cry features elicited by painful circumcision procedures and the perceived urgency of those cries. The authors found that individual crying bouts became shorter and more frequent as pain increased. Also, Bellieni et al. (2004) assessed differences in sound spectra of crying of term newborns in relation to different pain levels, and observed that as pain intensity increased, a stereotyped cry was produced, characterized by repeated cries separated by short, quieter intervals. The authors suggested that continuous repetition of the same sound signal may be an effective way of alerting the listener, possibly as a call for attention and help. Similarly, people with late-stage dementia (e.g., noncommunicative nursing home residents) who experience physical discomfort or pain typically display intense repetitive vocalization (Chanques et al., 2009; Cohen–Mansfield and Creedon, 2002; Kovach et al., 1999). Taken together, these findings indicate that a higher number of tonal segments or pulses may serve as a pain indicator.

### Shimmer Level

Shimmer is a measurement of F0 disturbance relates to the amplitude variation of the sound wave (i.e., variations of the loudness of a speaker’s voice). Typically, it can be analyzed under a steady voice production (e.g., producing a vowel continuously). The shimmer changes with the reduction of glottal resistance and mass lesions on the vocal folds and is correlated with the presence of noise emission and breathiness, with lower shimmer values represent more stable voice quality (i.e., smaller variation in amplitude). For adults, it is considered pathological voice for values higher than 3% (Teixeira et al., 2013).

Fuller and Horii (Fuller and Horii, 1986) suggested that stress arousal (e.g., pain-induced vocalization) which involves increases in the tension of striated muscles, in the rate and depth of breathing, and in airway diameter may alter the shimmer level. In the present study, pain related as well as no-pain related vocalizations were characterized by high (pathological) shimmer values, possibly reflecting the increased muscle tension and abnormal vibratory pattern of the vocal folds that characterizes individuals with CP [(Chen et al., 2018); For related findings in adults with IDD, see: (Icht, 2019)]. However, in pain related vocal expressions shimmer levels were significantly higher (14%) relative to no-pain related vocal expressions (11%). Results from the literature echo this finding, with higher shimmer levels were associated with pain (Raine et al., 2019; Salinas–Ranneberg et al., 2017).

### Other Acoustic Measures

Descriptive data indicate that pain related productions were characterized by several other acoustic traits, that is, longer duration, higher jitter and CPP values, and lower HNR and H1H2c values, and higher F0 mean and SD relative to no-pain related vocal productions, yet these differences were not statistically significant, possibly due to relatively small sample size and the high variability that characterizes individuals with IDD.

Reviewing the pertinent literature reveals similar findings, as pain vocalizations are typically longer in duration (Porter et al., 1986; Johnston and O’shaugnessy, 1987; Scheiner et al., 2006; Raine et al., 2019), have higher jitter levels (Tiezzi et al., 2004) and overall higher levels of roughness [irregular or chaotic vocal fold vibration; (Facchini et al., 2005; Koutseff et al., 2018; Tiezzi et al., 2004)] which associate with lower HNR values [see also Icht et al. (2018)]. As suggested by Tiezzi et al. (2004) (Kovach et al., 1999), pain causes a higher rigidity of the tissues in general, and of the vocal folds in particular, resulting in these latter acoustic features related to vocal quality (noise). In a similar vein, Facchini et al. (2005) posited that pain causes muscle contraction and an increase in lung pressure. These may lead to distortion of the oscillating system, namely large variations in vocal fold tension and tissues constants. Accordingly, an increase in pain induces a chaotic transition in vocal fold oscillation, so that pain related vocalizations associate with noise patterns.

As aforementioned, in the current study, pain related productions were also characterized by lower H1H2c values (non-significant). H1H2 measures the amplitude difference between the first harmonic (the fundamental frequency) and the second harmonic (the first multiple of the fundamental). H1–H2 serves as a measure of spectral tilt (or spectral slope), which is defined as the slope of least squares linear fit to the log power spectrum (Enflo, 2009), where a lower spectral tilt corresponds to a louder voice. H1–H2 is highly correlated with the degree of glottal constriction, and it has more cycle-to-cycle variability in dysphonic voices than in non-pathologic voices (Urberg–Carlson, 2013). Interestingly, lower H1–H2 has been associated with creaky voice, while higher H1–H2 occurs with breathy voice [(Gittelson et al., 2021; Keating and Esposito, 2006); see Figure 2]. This pattern may correspond to the current findings, as creakiness was found to be one of the most robust elements of expressions of pain and discomfort (Jenkins and Hepburn, 2015), possibly requiring less effort to produce, and a by-product of not fully opening one’s throat and engaging vocal folds.

Pathological voices, as well as pain related productions, are characterized by the addition of noise in the voice spectrum and aperiodicity. Quantification of noise in voice signals has been implemented using HNR, with dysphonic voices have lower values of this measure than non-pathologic voices (Madill et al., 2019). Alternatively, the Cepstral Peak Prominence (CPP) can be used as a reliable and sensitive measure of dysphonia. It can be measured from many types of signals (e.g., prolonged vowel, connected speech), and is calculated as the difference in amplitude between the cepstral peak and the corresponding value on the regression line directly below the peak. The more periodic the voice signal, the greater the degree of harmony, and the greater the value of the CPP (Hillenbrand et al., 1994). In the current study, no significant difference was found in CPP values between pain- and no-pain related productions (16 vs. 15 dB, respectively). Comparing these values to the CPP cutoff values recently reported by Murton et al. (2020) may hint to the absence of voice disorders in the current sample of participants. However, as CPP values can vary widely with different speaking tasks, languages, and computation algorithms, future studies may further assess this objective measure and its relationship to pain.

With regard to F0 measures, findings in the literature indicate that pain vocalizations are characterized with higher F0 mean and SD relative to no-pain related productions [e.g., Porter et al. (1986), Scheiner et al. (2006), Belin et al. (2008), Salinas–Ranneberg et al. (2017), Koutseff et al. (2018)], as found in the current study as well. According to Sisto et al. (2006), cry pitch is linked to vagus nerve tone (i.e., to the parasympathetic system). Stress causes a decrease in this tone, prompting increased tension in the vocal folds which are innervated by the vagus nerve.

Note, however, some studies failed to document correlations between F0 measures [and other acoustic measures, such as duration of crying) and pain scores [e.g., Johnston and Strada (1986)]. This inconsistency in the literature calls for further investigations. In addition, many of the above-mentioned studies were conducted within groups of neurotypical babies and infants, while the current investigation referred to adults with IDD, therefore relevance of these findings to the current population needs yet to be reevaluated. The current findings extend our knowledge on unique pain related vocal patterns to the special group of adults with severe IDD, again, highlighting the need and relevance of additional research in this field.

In sum, the current initial findings indicate vocal features (mainly number of pulses and shimmer level) as possible pain determinants in need of further investigation. These findings stress the need to consider individual vocal characteristics of people with IDD with the aim of improving pain management and avoiding pain underdiagnosis and under-treatment, particularly in non-verbal individuals with IDD.

### Limitations and Future Directions

Given the preliminary nature of this study, several limitations warrant discussion. First and foremost, the current sample of acoustic data was small, as the original sample was limited, and some recordings did not enable acoustic analysis. Future studies should be conducted with a larger sample size, enabling parametric statistical analysis, to enable generalization of the results.

In addition, the use of existing data (video recordings of a previous study) prevented a full acoustic analysis. For example, original recordings were performed using the video-camera built-in microphone with environmental variables and ethical issues determining its distance and positioning (e.g., the need to avoid filming the participant’s face, to prevent identification). Since the distance between the speaker and the camera was not controlled, variables of vocal intensity were not analyzed. In addition, the video recordings were conducted in daily situations in a noisy day-care center using the microphones embedded in the video camera, rather than in a sound-treated booth by a designated sensitive microphone. Noise and reverberation conditions in the room were not controlled or documented. Future research should be performed with professional recording equipment in more auditory controlled experimental setting and by implementing pre-determined professional guidelines. For example, a non-invasive contact microphones, which is insensitive to background noise (Carullo et al., 2013; Carullo et al., 2015) can be placed at the base of the neck during all day, to enable long-term voice monitoring. Future studies, specifically designed for voice in IDD, may include more controlled voice samples (e.g., sustained vowel/a/), to gage additional indices, such as CPP smoothed (CPPS) distribution (Castellana et al., 2018) as possible pain indicators.

Finally, it is important to note that the sample of participants was heterogeneous, and many background factors were not controlled, for example, IDD etiology and comorbidity. Future studies of larger and more homogeneous groups are warranted.

## Conclusion

Pain assessment of individuals with IDD, specifically those diagnosed with severe and profound IDD is extremely challenging. This difficulty might lead to under-detection of pain situations, thereby leading to under treatment of painful situations in the above-mentioned population. The preliminary findings presented by the current investigation suggest the possibility of using audio signals as possible pain monitoring and detection in individuals with IDD. These preliminary results necessitate further investigation.

## Data Availability Statement

The raw data supporting the conclusions of this article will be made available by the authors, without undue reservation.

## Ethics Statement

The studies involving human participants were reviewed and approved by the Institutional Review Board of Ariel University. Written informed consent to participate in this study was provided by the participants’ legal guardian/next of kin.

## Author Contributions

All authors listed have made a substantial, direct and intellectual contribution to the work, and approved it for publication.

## Conflict of Interest

The authors declare that the research was conducted in the absence of any commercial or financial relationships that could be construed as a potential conflict of interest.
